# Iris from Iridectomy Used as Spacer underneath the Scleral Flap: The Iridenflip Trabeculectomy Technique

**DOI:** 10.1155/2015/359450

**Published:** 2015-10-22

**Authors:** Veva De Groot, Liselotte Aerts, Stefan Kiekens, Tanja Coeckelbergh, Marie-José Tassignon

**Affiliations:** ^1^Department of Ophthalmology, University Hospital Antwerp, Wilrijkstraat 10, 2650 Edegem, Belgium; ^2^Department of Ophthalmology, Antwerp University, Universiteitsplein 1, 2610 Wilrijk, Belgium

## Abstract

*Purpose*. We describe a modified trabeculectomy technique in which the iris is used to prevent fibrosis of the scleral flap. *Material and Methods*. A retrospective case series of patients with medically uncontrolled open angle glaucoma underwent trabeculectomy. Instead of performing a classical iridectomy, the iris was used as spacer underneath the scleral flap. Postoperative management was identical to classical trabeculectomy, with suture removal and needling if necessary. Five of the patients underwent simultaneous phacoemulsification through a separate temporal corneal incision. Patients should have two-year follow-up. *Results*. Data of ten patients were analysed, two had a previous failed trabeculectomy, two had LTP, and one had a corneal transplantation. In 3 patients MMC 0,1 mg/mL was used. After one and two years mean IOP was, respectively, 13,1 and 12,1 mmHg. IOP ≤ 16 mmHg was reached in 90% of patients without pressure lowering medication. No major complications were seen; no abnormal inflammatory reaction and no deformation or dislocation of the pupil occurred. *Conclusion*. By using the iris from the iridectomy as spacer under the scleral flap, fibrosis of the scleral flap is no longer possible. This iridenflip trabeculectomy technique gives an excellent complete success rate (IOP ≤ 16 mmHg) of 90%. A larger study is currently being done.

## 1. Introduction

Surgical treatment of COAG can be very effective in reducing IOP; however success ratios are still not optimal. Trabeculectomy is the most frequently performed filtering procedure and many variants have been developed, but they often face failure due to conjunctival or scleral fibrosis [1]. Postoperative manipulations as needling and 5-FU injections are used to increase success rates. Peroperative application of mitomycin C is used to reduce fibrosis of tenon, conjunctiva, and scleral flap. Different kinds of resorbable or nonresorbable implants can be placed underneath the scleral flap to avoid fibrosis of the sclera.

We describe a technique in which the iris of the iridectomy is used as a spacer to prevent fibrosis of the scleral flap.

## 2. Material and Methods

Retrospective case series was as follows. Eleven consecutive patients with medically uncontrolled open angle glaucoma underwent trabeculectomy with the iridenflip technique. Outcome measures were IOP and postoperative complications. Patients should have at least two years of follow-up. There were no other exclusion criteria.

*Trabeculectomy technique* was similar to our classical technique, with the only difference that the iris from the iridectomy was not discharged but used as spacer underneath the scleral flap.

**Figure 1:**
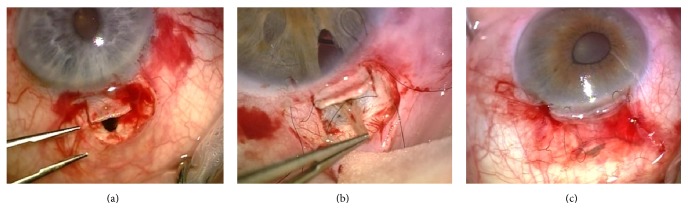
Surgical steps in different patients. (a) Iris flap extending beyond the scleral flap. (b) Iris grasped by forceps, pigment has already been removed. (c) Iris can be visible under the conjunctiva after closure.

Trabeculectomy was performed under retrobulbar anaesthesia. After a fornix based conjunctival flap, a rectangular scleral flap of 3 by 2,5 mm was made. A second triangular scleral flap is prepared up to Descemet's membrane and is removed completely including the trabeculectomy. Instead of performing a classical iridectomy, an angle based V-shaped iris incision is made. The iris base remains attached while the iris triangle is flipped onto the sclera. The length of the iris flap should be slightly longer than the scleral flap. The pigmented posterior iris epithelium is removed by gently touching it with a sponge. The scleral flap is closed with 2 releasable sutures' nylon 10/0 according to Khaw's technique [2]. The tip of the iris triangle should remain visible after closing the scleral flap (Figure 1). Conjunctiva and tenon are closed with 2 limbal sutures and 1 conjunctival suture vicryl 10/0. The anterior chamber was maintained during the procedure by injecting viscoelasticum through paracenteses just before resection of the second scleral flap. The viscoelasticum was not completely removed.

Postoperative treatment consisted of atropine 2% 3 times daily and tobramycin combined with dexamethasone 4 times daily.

*Postoperative management* was similar as in classical trabeculectomy. Patients were seen at day 1, every week for 5 weeks, and then after 2 weeks and every 3 months thereafter. If IOP was too high manual pressure was applied to the eye to stimulate evacuation of aqueous or viscoelasticum towards the bleb. Suture removal was performed if pressure remained above 15 mmHg after one week or after 1 month in all patients.

Topical steroids were tapered slowly over 3 to 4 months if pressure was good or increased if the bleb vascularisation increased or bleb size diminished or if pressure raised above 15 mmHg. Atropine was stopped after 2 weeks, unless there was persistent hypotony.

Subconjunctival 5-FU injection was planned if conjunctival corkscrews vessels were seen.

Complete success rate was defined as IOP ≤ 16 mmHg without any topical or systemic pressure lowering medication.

## 3. Results

### 3.1. Patient Data

Eleven patients with COAG underwent trabeculectomy with the iridenflip technique. Five of them had a combined phacotrabeculectomy. None of them had a narrow angle. One patient was lost to follow-up after 5 months, with a pressure of 10 mmHg, and was excluded.

Ten patients were included in the analysis. Patient characteristics are shown in Table 1. All had COAG with pressure between 19 and 40 mmHg (with a mean of 27,7 mmHg) on maximal topical therapy.

Male/female ratio was 1/1 and age ranged from 52 to 85 years (with a mean of 72,9 years).

All patients were on topical pressure lowering medications, ranging from 1 to 4 (mean of 2.7 medications). Visual field MD defects ranged from 1,5 to −22,8 dB (mean −13,28 dB). Two patients had a previous failed trabeculectomy and two had LTP. One patient had a history of corneal transplantation.

In 3 patients (30%) MMC 0,1 mg/mL was applicated during 1 minute because of a scarred conjunctiva or a low target pressure.

### 3.2. Postoperative Complications

Visual acuity was stable or increased in the combined procedures.

Postoperative complications are shown in Table 2.

One patient had a wound leak on the first day, which did not heal with a contact lens and was closed by an additional suture. One patient had hypotony with choroidal effusion in one quadrant the first 2 weeks, which disappeared spontaneously. One patient had a small hyphema the first 2 weeks.

In one patient with a low target pressure a needling revision with MMC was performed after 7 weeks because of IOP of 18 mmHg.

5-FU was never needed.

**Figure 2:**
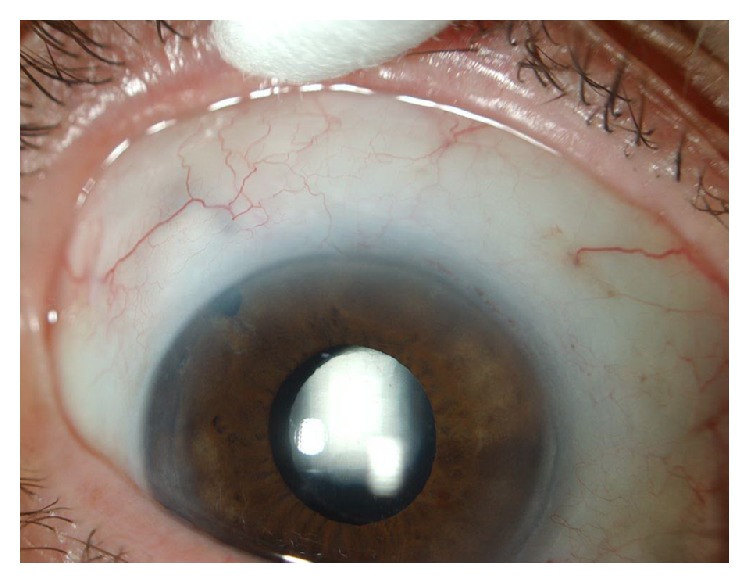
Postoperative view of trabeculectomy with iridenflip and diffuse bleb superotemporal.

No late complications were seen. No abnormal inflammatory reaction and no deformation or dislocation of the pupil occurred (Figure 2). The pupil was round and well centred in all our cases.

### 3.3. Trabeculectomy Outcome

After one year mean pressure was 13,1 mmHg, nine patients had IOPs ranging from 10 to 16 mmHg and one patient had an IOP of 19 mmHg (patient with combined phacotrabeculectomy in which topical medication was started).

**Figure 3:**
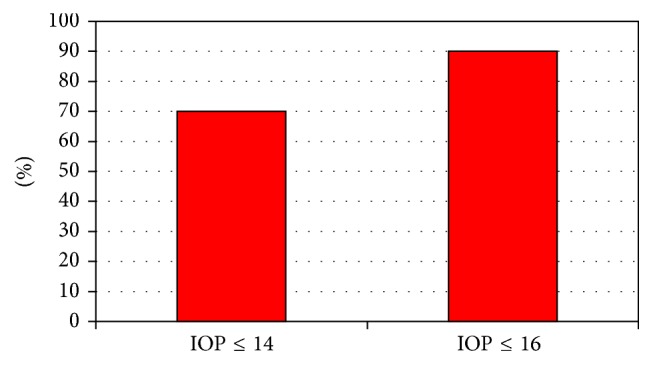
Percentage of patients with complete success (without medication) for a given target IOP in mmHg after 2 years of follow-up.

A pressure of 16 or lower was reached in 90% of patients without any pressure lowering medication after one year. In 70% IOP was even 14 or lower without medication (Figure 3).

After two years mean pressure was 12,1 mmHg, ranging from 6 to 16 mmHg, with identical success rates.

## 4. Discussion

The initial results of this iridenflip trabeculectomy technique are very encouraging.

Our small series demonstrated a complete success ratio of 90% in achieving IOP ≤16 mmHg after one and two years and in 70% IOP was ≤14 mmHg. Mitomycin C was used in 30% of the patients.

Comparing trabeculectomy outcomes between different studies is difficult due to a variety in defining success rates and use of antimetabolites.

The trabeculectomy outcome study group [3] reported a complete success rate of 78% in reaching IOP ≤18 mmHg in 428 patients after 2 years, and antifibrotic agents were used in 93% of all cases. Cillino et al. [4] reported a complete success rate of 55% in reaching a target IOP ≤17 mmHg after trabeculectomy (*n* = 18) and 33% after phacotrabeculectomy (*n* = 15) without antimetabolites with a mean follow-up of 22,5 months.

Our complete success ratio can compete with the qualified success (with medication) of some larger studies. In the trabeculectomy arm of the TvT study [5] they report a qualified success rate of 86,5%, defined as IOP < 21 mmHg and >20% below baseline.

But we like to stress that our series of 10 patients is too small to draw conclusions on efficacy.

Current trabeculectomy technique still faces failure due to conjunctival or scleral fibrosis. It is known from older reports of the iridencleisis procedure that iris tissue can provide a long lasting fistulisation through the sclera [6, 7]. After performing trabeculectomy and iridectomy, the small strand of iris tissue with its furrows and crypts is discharged. Instead of using resorbable or nonresorbable foreign material as spacer underneath the scleral flap, the iris is an excellent alternative. Fibrosis at the level of the scleral flap is not possible if the iris is interposed. Since the tip of the iris reaches further than the scleral flap, the aqueous is drained through the sclera up to the subtenon and subconjunctival space. At the tenon or conjunctival level fibrosis is possible as in all other filtering procedures. Postoperative management of conjunctival fibrosis remains necessary. Our technique may have the advantage of decreasing scleral flap fibrosis.

Following these first 10 cases, no abnormal inflammation or other problems were seen, compared to classical trabeculectomy. We had no higher incidence of postoperative manipulations. In one patient a needling revision was necessary in order to achieve a low target pressure.

This technique is completely different from the old iridencleisis technique.

In the original Holth's iridencleisis the iris prolapsed through the sclerotomy and was grasped with two forceps at the pupil and torn [8]. The two iris pillars were pulled through the sclerotomy giving rise to U shaped pupil [9]. Pressure lowering effect was often very good, with reports of up to 90% of patients with chronic glaucoma being controlled [10]. And this is without using antimetabolites.

In later modifications only the peripheral iris was prolapsed and incarcerated in the sclerotomy [11], leaving the pupil intact, but with a lower success rate [12] since aqueous flow could be trapped in the fold of the iris. To avoid this also the iris root was ruptured while pulling the iris to one side of the sclerotomy, referred to as the lobe-like iridencleisis [13].

The major drawback of all techniques was a complete distortion of the pupil with pear shaped corectopia pointing towards the 12 o'clock position, often with an upward displacement of the pupil. In case the iris sphincter was completely sectioned it resulted in a superior coloboma [8].

In all our cases the pupil was round and well centred, with normal pupil reflex. When looking with the slit lamp an iridectomy is visible, but the deference with a classical iridectomy cannot be seen. Only with gonioscopy the iris base can be seen turning into the trabeculectomy window.

A second reason why iridencleisis may have fell out of use is the possible risk of sympathetic uveitis, although only a total of 4 cases of sympathetic ophthalmia following iridencleisis have been reported [7]. In the same first half of the 20th century, at least 652 cases of sympathetic ophthalmia were reported, following trauma, cataract surgery, and many other intraocular surgeries [7]. Sourdille, however, did never encounter sympathetic ophthalmia in his series of 236 iridencleisis procedures and stated that iridencleisis is no more dangerous than other fistulising operations [14]. In later reports presenting modified iridencleisis procedures, the issue of sympathetic ophthalmia is not even mentioned anymore [15].

In our series we did not encounter any iritis or uveitis. In view of this historical threat of sympathetic ophthalmia, we reduce the amount of pigmented uveal tissue below the conjunctiva, by gently removing the posterior layer of the iris epithelium. The second reason to remove the pigmented layer was to avoid postoperative spread of these pigmented cells below the conjunctiva due to the aqueous flow. This can create a visible pigmentation which might be mistaken for a subconjunctival nevus or melanoma.

## 5. Conclusion

In this study the iris from the partial iridectomy was used as spacer underneath the scleral flap, in order to prevent fibrosis of this scleral flap. The data show that no major complications were observed within the short observation period of 2 years.

A major limitation of the study is the small number of patients and the absence of a control group. A larger RCT is needed to substantiate the alleged benefits.

## Figures and Tables

**Table 1:** Preoperative patient demographics.

Patient characteristics	*N* = 10 or mean
Male/female ratio	1/1
Age	72,9 years (range 52 to 85)
MD defects	−13,28 (range 1,5 to −22,8)
IOP preop. on max therapy	27,7 mmHg (range 19 to 40)
Number of topical IOP lowering meds	2,7 (range 1 to 4)
Previous LTP	2/10
Previous trabeculectomy	2/10
Previous corneal transplant	1/10

**Table 2:** Postoperative complications.

Postop. complications	*N* = 10
Wound leak first day	1
Small hyphema during 2 weeks	1
Hypotony + small choroidal effusion (2 weeks)	1
Bleb failure	1
